# An atypical case of jejunal gastrointestinal stromal tumour presenting as chronic anaemia

**DOI:** 10.1093/jscr/rjag069

**Published:** 2026-02-17

**Authors:** Shadin S A Abushara, Dawood D M Mahmood, Ahmed Haidaran

**Affiliations:** General Surgery Department, Galway University Hospital, Newcastle Road, Galway City, H91YR71, Ireland; General Surgery Department, Galway University Hospital, Newcastle Road, Galway City, H91YR71, Ireland; General Surgery Department, Cork University Hospital, Wilton Street, Cork City, T12DC4A, Ireland

**Keywords:** jejunal GIST, chronic anaemia, CT angiography, laparoscopic resection, low-risk tumour

## Abstract

Gastrointestinal stromal tumours (GISTs) are rare mesenchymal neoplasms, most commonly arising in the stomach or small intestine. Jejunal GISTs are rare and may pose diagnostic challenges. We report a 44-year-old woman with a 3-year history of chronic unexplained iron deficiency anaemia and negative endoscopy. Computed tomography angiography suggested a jejunal lesion. Laparoscopy identified a well-circumscribed jejunal mass, which was resected. Histopathology confirmed a low-risk GIST. This case highlights the importance of considering small bowel pathology in persistent anaemia and the role of cross-sectional imaging or capsule endoscopy in timely diagnosis.

## Introduction

Gastrointestinal stromal tumours (GISTs) are rare mesenchymal neoplasms, representing ~5% of all soft tissue sarcomas, as they are considered to originate from smooth muscle. It is most commonly found in the stomach and small intestine. Jejunal GISTs are particularly uncommon and account for a small subset of small bowel neoplasms [1]. These tumours can be asymptomatic. However, they typically present with gastrointestinal (GI) bleeding, abdominal pain, or obstruction. Presentation as chronic iron deficiency anaemia without overt GI symptoms is atypical and may delay diagnosis.

## Case report

**Figure 1:**
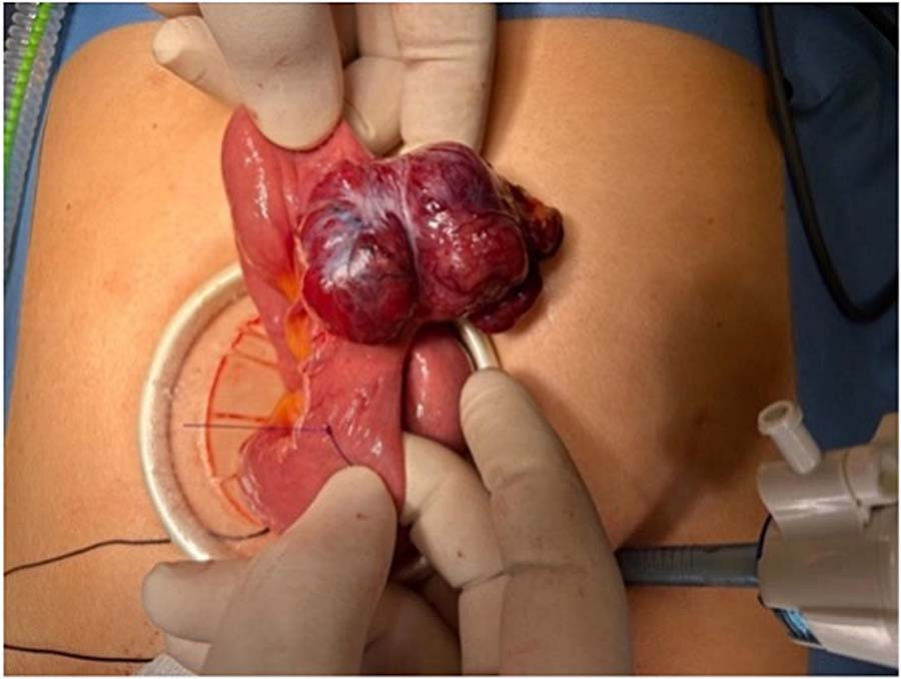
Laparoscopic-assisted delivery of a lobulated jejunal mass arising from the antimesenteric border, consistent with a gastrointestinal stromal tumour.

**Figure 2:**
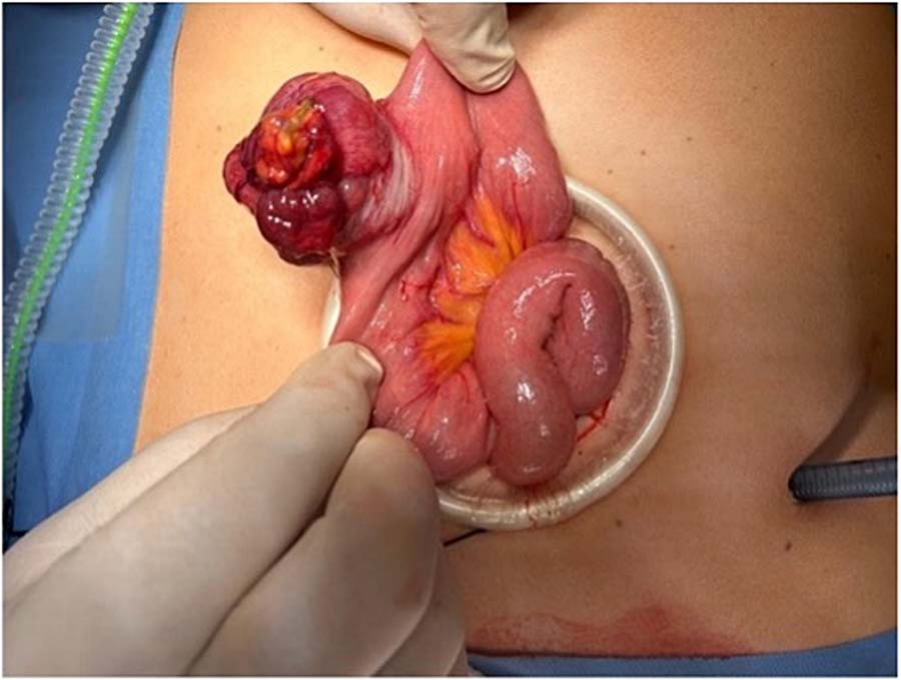
Exposure of the jejunal segment through a mini-laparotomy, demonstrating the exophytic tumour prior to segmental resection.

A 44-year-old married woman presented with a 3-year history of anaemia. She was seen by various specialties throughout this period, had multiple investigations performed, but no underlying cause was identified. Despite iron supplementation, her haemoglobin (Hb) levels remained between 8 and 10 g/dl. Upper gastrointestinal endoscopy (OGD) and colonoscopy were normal. She received triple therapy for *H. pylori* following a negative colonoscopy and OGD. However, this was also unsuccessful in managing her anaemia. The patient denied any prior medical illness, per rectal (PR) bleeding, or weight loss. In May 2023, she was admitted to the hospital under medical care for weakness and severe anaemia. During her hospitalization, she developed PR bleeding, and her Hb dropped to 5 g/dl, prompting a surgical consultation on 15 May 2023. CT angiography revealed a possible small bowel lesion in the jejunum without signs of active bleeding. On 16 May 2023, laparoscopic exploration identified a well-defined, egg-sized lesion in the jejunum (Figs 1 and 2). A 10 cm midline incision was made, and a segmental resection of the jejunum with a 5 cm safety margin was performed. Side-to-side hand-sewn anastomosis was completed using 3/0 PDS suture.

The patient had an uneventful postoperative recovery, tolerating a liquid diet after 48 hours. She transitioned to a normal diet and was discharged on postoperative day 4. Histopathological examination confirmed a non-obstructing GIST tumour measuring 4.1 cm in its maximum dimension.

A MDT discussion determined that no further imaging follow-up was necessary due to the tumour’s low-risk classification. However, at the patient’s request, additional CT thorax, abdomen, and pelvis (CT TAP) scans were performed at 1 and 2 years post-surgery, both showing no recurrence or metastasis. Her Hb levels normalized following surgery and remained stable in subsequent blood tests.

## Discussion

GISTs are rare mesenchymal neoplasms of the gastrointestinal tract, accounting for ~0.1%–3% of all GI malignancies [2]. They most commonly arise in the stomach (60%) and small intestine (30%), with jejunal GISTs comprising a relatively small proportion of cases [3]. Clinical presentation varies significantly and may include abdominal pain, gastrointestinal bleeding, or incidental detection on imaging. As demonstrated in this case, chronic iron deficiency anaemia as a sole manifestation, without overt bleeding or constitutional symptoms, is unusual and contributes to diagnostic delay.

Our patient in this case experienced a prolonged course of unexplained anaemia over 3 years, despite extensive evaluation, including normal upper and lower endoscopies and empirical *Helicobacter Pylori* eradication therapy. The absence of visible gastrointestinal bleeding or weight loss likely contributed to a low index of suspicion for small bowel pathology. It was not until a severe exacerbation of her anaemia accompanied by overt rectal bleeding that more targeted imaging, specifically CT angiography, was performed, leading to the identification of a suspicious lesion in the jejunum.

Small bowel lesions such as GISTs are challenging to diagnose using conventional diagnostic modalities. Routine endoscopy cannot access the mid-small bowel, and even capsule endoscopy may miss submucosal masses. CT angiography, in this case, proved valuable by localizing a possible mass, despite no signs of active bleeding. This reflects the growing utility of cross-sectional imaging in obscure gastrointestinal bleeding or persistent iron deficiency anaemia of unknown origin [4].

Laparoscopic exploration facilitated diagnosis and curative treatment. Surgical resection remains the mainstay treatment of choice for localized GISTs, with complete resection and negative margins offering excellent prognosis, especially in low-risk tumours [5]. The surgical approach in this case, laparoscopic-assisted resection with a hand-sewn anastomosis, was both effective and minimally invasive, contributing to an uneventful and prompt recovery.

Histopathological examination confirmed a non-obstructing GIST tumour measuring 4.1 cm in its maximum dimension. A multidisciplinary team (MDT) discussion concluded no further surveillance imaging was necessary, given the tumour’s low-risk classification based on size and presumed mitotic index. However, in line with the patient’s preference, follow-up CT TAP scans were performed at 1 and 2 years postoperatively, showing no recurrence or metastasis. The patient's haemoglobin levels normalized following surgery and remained stable, further confirming the lesion as the source of chronic blood loss.

Histologically, GISTs are characterized by spindle or epithelioid cells and are usually positive for CD117 (c-KIT) or DOG1 on immunohistochemistry [6]. Risk stratification based on tumour size, mitotic rate, and location is essential to guide the need for adjuvant therapy and follow-up. Low-risk lesions such as this case typically require no adjuvant treatment and have a favourable prognosis.

## Conclusion

Jejunal GIST should be considered in patients with unexplained chronic anaemia and negative upper and lower endoscopies. Submucosal masses with or without bleeding may be missed on routine investigations; advanced imaging modalities, such as CT angiography or capsule endoscopy, should be considered early, especially when small bowel bleeding is suspected. Complete surgical resection remains the mainstay of treatment, with minimally invasive approaches offering excellent outcomes.
